# S-nitrosylated and non-nitrosylated COX2 have differential expression and distinct subcellular localization in normal and breast cancer tissue

**DOI:** 10.1038/s41523-020-00204-6

**Published:** 2020-11-24

**Authors:** Sonali Jindal, Nathan D. Pennock, Alex Klug, Jayasri Narasimhan, Andrea Calhoun, Michelle R. Roberts, Rulla M. Tamimi, A. Heather Eliassen, Sheila Weinmann, Virginia F. Borges, Pepper Schedin

**Affiliations:** 1 Department of Cell, Developmental & Cancer Biology, Oregon Health & Science University, 2720 SW Moody Ave., Mailing Code: KR-CDCB, Portland, OR 97201 USA; 2 Knight Cancer Institute, Oregon Health & Science University, 2720 SW Moody Ave., Mailing Code: KR-ADM, Portland, OR 97201 USA; 3 Department of Dermatology, Massachusetts General Hospital, 50 Staniford Street, Suite 200, Boston, MA 02114 USA; 4 Channing Division of Network Medicine, Department of Medicine, Brigham and Women’s Hospital and Harvard Medical School, Boston, MA 02115 USA; 5 Department of Epidemiology, Harvard T.H. Chan School of Public Health, Boston, MA 02114 USA; 6 Center for Health Research, Kaiser Permanente Northwest, 3800 N. Interstate Ave., Portland, OR 97227 USA; 7 School of Medicine, Division of Medical Oncology, University of Colorado Anschutz Medical Campus, MS8117, RC-1S, 8401K, 12801 E 17th Ave., Aurora, CO 80045 USA; 8 Young Women’s Breast Cancer Translational Program, School of Medicine, Division of Medical Oncology, University of Colorado Anschutz Medical Campus, MS8117, RC-1S, 8401K, 12801 E 17th Ave., Aurora, CO 80045 USA

**Keywords:** Breast cancer, Cancer microenvironment, Nitrosylation, Tumour biomarkers, Nitrosylation

## Abstract

Immunohistochemical (IHC) staining in breast cancer shows both gain and loss of COX2 expression with disease risk and progression. We investigated four common COX2 antibody clones and found high specificity for purified human COX2 for three clones; however, recognition of COX2 in cell lysates was clone dependent. Biochemical characterization revealed two distinct forms of COX2, with SP21 recognizing an S-nitrosylated form, and CX229 and CX294 recognizing non-nitrosylated COX2 antigen. We found S-nitrosylated and non-nitrosylated COX2 occupy different subcellular locations in normal and breast cancer tissue, implicating distinct synthetic/trafficking pathways and function. Dual stains of ~2000 breast cancer cases show early-onset breast cancer had increased expression of both forms of COX2 compared to postmenopausal cases. Our results highlight the strengths of using multiple, highly characterized antibody clones for COX2 IHC studies and raise the prospect that S-nitrosylation of COX2 may play a role in breast cancer biology.

## Introduction

The cyclooxygenase enzyme COX2, a key mediator of tissue inflammation via prostaglandin production, has been investigated extensively as a cancer biomarker and therapeutic target^1–5^. Data supporting pro-tumorigenic roles for COX2 include robust preclinical studies identifying COX2 as an oncogene^6–9^; the demonstration that NSAID-based COX2 blockade inhibits cancer progression in preclinical models^10–12^ and epidemiologic studies showing that NSAID use correlates with reductions in colon and breast cancer risk^13–16^. However, prospective clinical trials utilizing aspirin or celecoxib therapy for the prevention, recurrence, and treatment of colon^17–21^ or breast cancer^22–24^ show variable results. Further, within the breast cancer field, disparate results of COX2 immunohistochemical (IHC) studies call into question the reliability of COX2 as a breast cancer biomarker or therapeutic target^25–31^.

In colon, where COX2 is firmly established as a tumor promoter, IHC studies on formalin-fixed paraffin-embedded (FFPE) tissue report minimal COX2 levels in normal epithelium, and increased COX2 in at-risk epithelium and invasive cancer^32–34^. While some breast cancer studies corroborate these results^35–39^, others show loss of COX2 in invasive disease compared to adjacent normal tissue, even when the same antibody clones are used^31,40–42^. One explanation for these divergent results may be methodological, as no standardized approach to COX2 IHC detection has been adopted. For example, one concern regarding αCOX2 antibodies is cross-reactivity, as COX1 is closely related to COX2, with 65% amino acid sequence homology and near-identical catalytic sites^43^.

In this study, we investigated the effect of antibody clone selection on COX1 and COX2 recognition, focusing on four commonly utilized αCOX2 clones. We validated three clones, SP21, CX229, and CX294 as highly specific for COX2 protein. Unexpectedly, we found these antibody clones differently recognized COX2 in COX2 positive cell lysates, in histologically normal breast tissue, and in breast and colon cancer tissues. We found these distinct staining patterns are due to antibody specificity for S-nitrosylated and non-nitrosylated forms of COX2. Further, we demonstrate distinct subcellular localization of COX2 based on S-nitrosylation. In summary, these studies infer distinct regulation and function of COX2 based on S-nitrosylation and highlight the strengths of interrogating COX2 with multiple, validated antibody clones.

## Results

### COX2 antibody validations

**Fig. 1:**
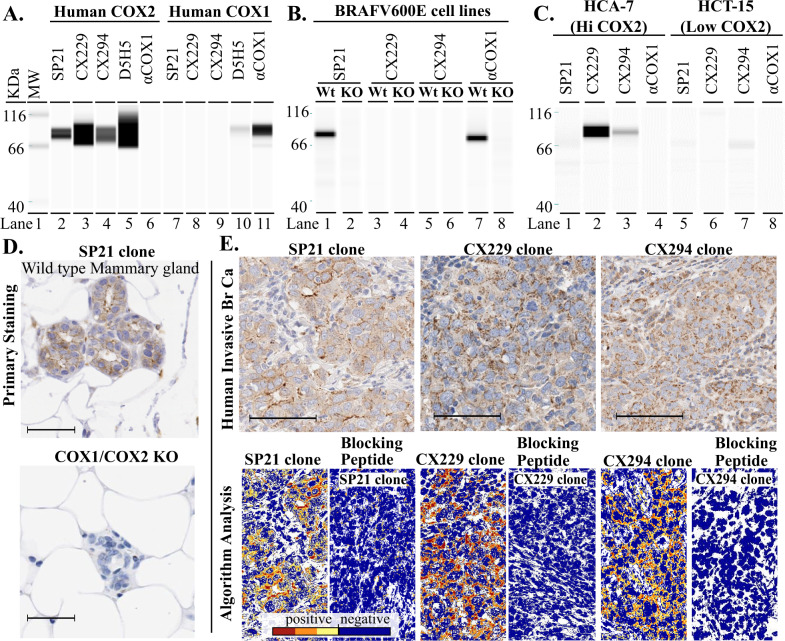
αCOX2 specificity of four distinct antibody clones. **A** Western blot analysis for αCOX2 clones SP21, CX229, CX294, and D5H5 against recombinant human COX2 and COX1 protein show high specificity of clones SP21, CX229, and CX294 for COX2 protein (lanes 2–4 and lanes 7–9). Clone D5H5 shows strong reactivity to COX2 protein, but also some reactivity to COX1 protein (lanes 5 and 10). Lane A11 is the COX1 protein/αCOX1 antibody positive control. **B** Mouse melanoma BRAFV600E cell lysate is recognized by SP21 in wild-type (Wt) cells, but not in COX1/COX2 KO cells (lanes 1 and 2). CX229 and CX294, made against human COX2, do not show reactivity to mouse COX2+ cell lysates (lanes 3 and 5). **C** Clones SP21, CX229, and CX294 were probed against human cell lysates with high (HCA-7) and low (HCT-15) COX2 expression. SP21 did not show reactivity to HCA-7 cell lysate (lane 1). CX229 and CX294 show reactivity to HCA-7 cell lysate (lanes 2 and 3). All three clones show minimal to no reactivity to HCT-15 cell lysates (lanes 5–7). **D** FFPE tissue stained using SP21 shows staining in the mammary epithelium of Wt, but not in COX1/COX2 KO mice. **E** Human breast cancer tissue stained for SP21, CX229, and CX294 show robust signal in the tumor cells (upper panels, brown stain). Quantitative algorithmic analysis shows positive (orange and red) and negative (yellow and blue) signal for SP21 and CX229 in human breast cancer tissue (lower left panels). COX2 epithelial signal with SP21, CX229, and CX294 is blocked using a COX2-specific blocking peptide (lower second, fourth, and sixth panels). Scale bar for all images is 50 µm.

We assessed four commonly utilized αCOX2 antibody clones, SP21, CX229, CX294, and D5H5 (Table 1) for COX2 specificity to recombinant human COX1 and COX2 proteins. We found all four antibodies recognized recombinant human COX2 protein (Fig. 1A, lanes 2–5). Only D5H5 showed weak reactivity to human COX1 (Fig. 1A, lane 10) and was eliminated from further evaluation.

**Table 1:** Comparison of structural properties of two COX2 antibodies.

Designated Clone	SP21	CX229	CX294	D5H5
Epitope	Rat COX2, C-terminus AA sequence 513–604	Human COX2, C-terminus AA sequence 580–599	Human COX2, C-terminus AA sequence 580–598	Human COX2 protein residues surrounding AA sequence 93–123
Host for antibody preparation	Rabbit	Mouse	Mouse	Rabbit
Clonality	Monoclonal	Monoclonal	Monoclonal	Monoclonal
Epitope COX1 overlap	48AA	2AA	2AA	17AA
Vendor Company (catalog #)	Thermo Scientific (RM-9121-R7)	Cayman Chemicals (160112)	Dako (M3617)	Cell Signaling (12282)

We next assessed specificity of SP21, CX229, and CX294 to detect COX2 protein in cell lysates from mouse melanoma BRAFV600E cells with wild type (Wt) or genetically deleted COX1/COX2 (KO)^44^. SP21 detected COX2 protein in Wt but not in KO cells (Fig. 1B, lanes 1 and 2). CX229 and CX294 did not detect murine COX2 (Fig. 1B, lanes 3 and 5), consistent with reported human specificity for these clones. To assess antibody reactivity to human COX2 protein, we utilized human colon cancer cell lines with high (HCA-7) and low (HCT-15) COX2 expression^45,46^. As anticipated, CX229 and CX294 detected COX2 protein in the high COX2-expressing HCA-7 cells (Fig. 1C, lanes 2 and 3), but not in the low-expressing HCT-15 cells (Fig. 1C, lanes 6–7). Unexpectedly, SP21 did not detect COX2 protein in the high COX2-expressing HCA-7 cell lysate (Fig. 1C, lane 1), even though SP21 robustly detects human recombinant COX2 protein (Fig. 1A, lane 2).

We next assessed for COX2 antibody specificity in FFPE tissues. Because SP21 recognizes murine COX2, we utilized genetically modified mouse models to confirm antibody specificity. We found that SP21 recognized murine COX2 in mouse mammary epithelial cells in Wt, but not in COX2 KO glands (Gift from Christopher Rivard, University of Colorado, Denver), confirming specificity (Fig. 1D). Next, a previously validated COX2 positive human breast cancer case^31^ was selected to assess SP21, CX229, and CX294 staining. All three clones stained this COX2 positive human control tissue (Fig. 1E). We next demonstrated SP21, CX229, and CX294 specificity for COX2 by confirming that the majority of antibody signal was lost with the addition of a COX2-specific blocking peptide (Fig. 1E). All three COX2 clones showed high specificity and sensitivity for COX2 in FFPE tissues.

### S-nitrosylated and non-nitrosylated forms of COX2

**Fig. 2:**
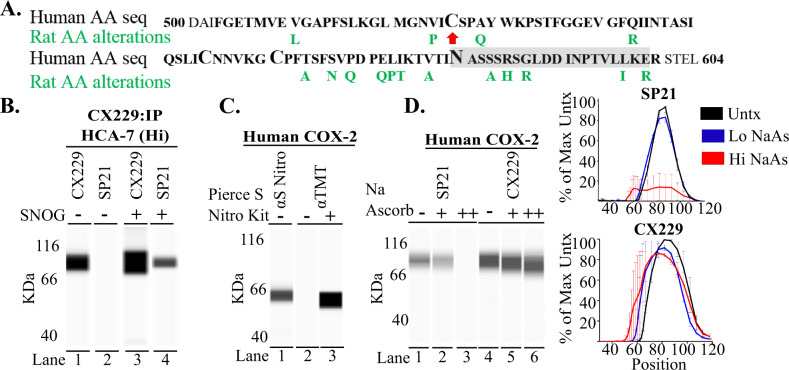
SP21 recognizes S-nitrosylated COX2. **A** Human and rat amino acid (AA) sequence of PTGS2 gene region used as immunogens (SP21 = black bold and CX229 = gray box). Potential posttranslational modification (larger font size) S-nitrosylation site is seen at cysteine 526 (red arrow), disulfide bond sites at AA 555 and 561, and a glycosylation site at AA 580. **B** COX2 immunoprecipitation (IP) of HCA-7 cell lysate using CX229 is recognized by CX229 (lane 1), but not SP21 (lane 2). On biochemical S-nitrosylation of the CX229 IP, SP21 regains reactivity to COX2 (lane 4). **C** Western blot analysis confirms recombinant human COX2 protein contains the S-nitrosylated form of COX2 as detected by a pan-nitrosylation specific monoclonal antibody (lane 1), as well as by assessment of nitrosylation modification using the Pierce S-nitrosylation kit and the anti-TMT antibody (lanes 2 and 3). **D** Western blot analysis confirms dose-escalating Na ascorbate treatment (−, +, ++) used for de-nitrosylation of recombinant human COX2 protein results in dose-dependent loss of SP21 signal (lanes 1–3). No or minimal loss of CX229 reactivity was observed after Na ascorbate treatment (lanes 4–6). Quantitation of COX2 western blots from three separate Na ascorbate experiments (right panel electrophoretograms).

Given the high specificity and sensitivity of SP21, CX229, and CX294 for COX2 protein, it is unclear why SP21 would not recognize COX2 in the COX2 high-expressing HCA-7 cells (Fig. 1C, lane 1). To address this question, we examined the amino acid sequences used as immunogens (Fig. 2A) for SP21, CX229, and CX294 antibody generation. We found a posttranslational modification site for S-nitrosylation at Cys-526 only in the SP21 immunogen (Fig. 2A, red arrow). CX229 and CX294 were made using essentially identical amino acid sequences. Since they similarly recognized COX2 in human colorectal cancer cell lines by western blot and have essentially identical staining in a breast cancer tissue microarrays (TMAs; *n* = 56, Supplementary Fig. 1 and Supplementary Table 1), we focused our subsequent biochemical analyses using SP21 and CX229. To address whether SP21 preferentially recognizes S-nitrosylated COX2, we employed the strategy of biochemically adding and removing nitric oxide (NO) moieties to COX2 protein, and then assessing antibody recognition by western blot. To obtain a source of COX2 that is differentially recognized by SP21 and CX229, and suitable for S-nitrosylation modifications, we performed COX2 immunoprecipitation of HCA-7 cell lysates using CX229, as CX229 recognizes COX2 in this cell line (as does CX294), whereas SP21 does not (Fig. 1C, lane 2 vs. lane 1). As expected, CX229 recognizes the COX2 protein immunoprecipitated by the CX229 antibody (Fig. 2B, lane 1), while CX229-immunoprecipitated COX2 was undetected by SP21 (Fig. 2B, lane 2). Since the SP21 immunogen includes the putative COX2 S-nitrosylation site, we reasoned that HCA-7 COX2 protein is non-nitrosylated, and that S-nitrosylation might convert HCA-7 COX2 to an SP21-recognizable form. To test this idea, the above CX229 immunoprecipitated COX2 was S-nitrosylated by incubation with S-nitrosoglutathione (SNOG)^47^. We found that SP21 detected HCA-7 COX2 only after incubation with SNOG (Fig. 2B, lane 4), which is consistent with SP21 specifically recognizing an S-nitrosylated form of COX2.

To determine if SP21 antibody signal is lost with de-nitrosylation of COX2 protein, as predicted if SP21 is specific for S-nitrosylated COX2, we next performed de-nitrosylation assays. Because recombinant human COX2 is recognized by SP21, suggesting S-nitrosylation (Fig. 1A, lane 2), we first determined if the recombinant human COX2 protein is S-nitrosylated by using a pan-nitrosylation-specific monoclonal antibody (Fig. 2C, lane 1), as well as a commercial (Sigma) biochemical detection kit for S-nitrosylation (Fig. 2C, lanes 2 and 3). Both methods demonstrate that purified recombinant human COX2 contains S-nitrosylated COX2. We then utilized sodium (Na) ascorbate to de-nitrosylate recombinant human COX2, utilizing a methodology previously reported for mouse cell lysates^48^. Na ascorbate treatment resulted in a dramatic dose-dependent decrease in SP21 signal (Fig. 2D, lane 3, and upper electrophoretogram). In contrast, de-nitrosylation of recombinant COX2 did not significantly reduce the CX229 signal (Fig. 2D, lane 6, and lower electrophoretogram). These western blot assay data are consistent with SP21 specifically recognizing S-nitrosylated COX2, whereas CX229 signal appears independent of S-nitrosylation.

### S-nitrosylated and non-nitrosylated COX2 staining patterns in human breast and colon cancer tissue

**Fig. 3:**
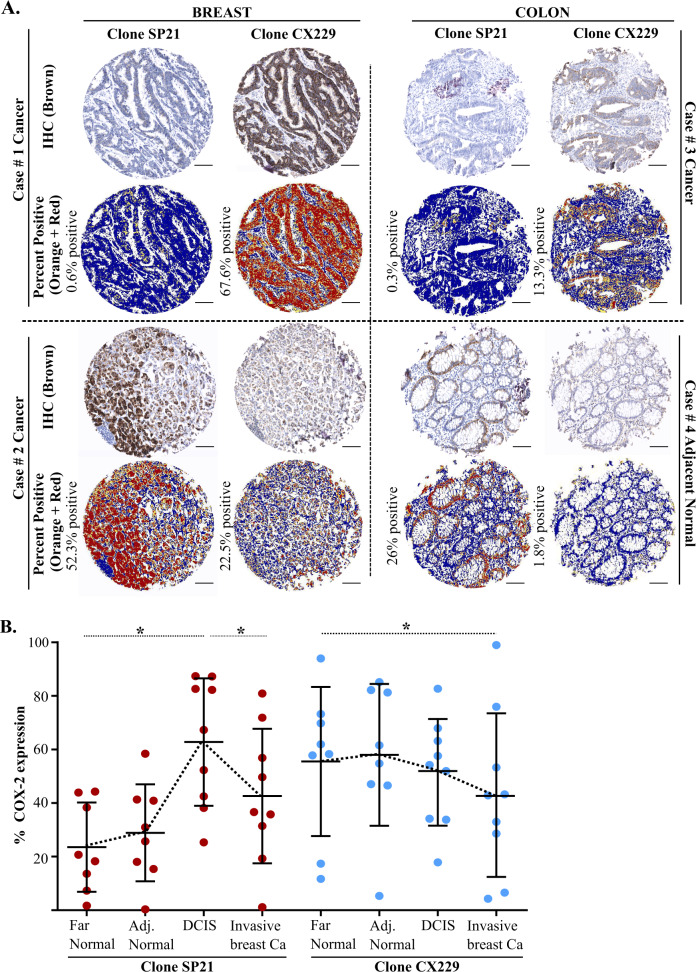
Two αCOX2 clones show differential pattern of staining. **A** TMA cores from breast and colon cancer cases show preferential CX229 (cases #1 and #3) or preferential SP21 staining (cases #2 and #4). Algorithmic analysis for each TMA core shows COX2 positive signal (orange and red) compared to negatively stained tissue (blue). **B** Sequential sections of individual cases were stained for SP21 (red) and CX229 (blue), and evaluated for COX2 expression in far and adjacent normal, DCIS, and invasive breast cancer tissue (*n* = 9). SP21 shows highest expression in the DCIS lesions. Clone CX229 shows highest COX2 expression in normal far and adjacent breast epithelium, with decreased COX2 expression in invasive cancer (*P values: ≤ 0.05). Scale bar for all images is 100 µm.

We next sought to determine if the S-nitrosylation state of COX2, as detected by SP21 and CX229, could result in disparate staining results in cancer tissue. To this end, we stained sections of human breast and colon TMAs, with 52 and 53 cores, respectively, using routine chromogen-based IHC methods. In breast TMAs, 2% of the cores stained preferentially with SP21, whereas 35% of the cores stained preferentially with CX229, with little to no overlap between staining patterns (Fig. 3A and Supplementary Table 2). Further, colon TMAs also had differential staining patterns for SP21 and CX229, suggesting relevance beyond breast (Fig. 3A and Supplementary Table 2). While unique staining patterns for SP21 were anticipated based on its specificity for S-nitrosylated COX2, the fact that CX229 antibody signal did not overlap with the SP21 signal was unanticipated, and strongly suggests that SP21 and CX229 recognize distinct forms of COX2.

### SP21 and CX229 show different staining trends in a breast cancer tissue cohort

We next addressed antibody staining patterns in a small (*n* = 9), but somewhat rare, breast tissue cohort that contained far normal (>1 cm distant form tumor), adjacent normal (>0.4 cm distant from tumor), ductal carcinoma in situ (DCIS), and invasive tumor on a single slide. In this cohort, the SP21 staining pattern showed increased COX2 expression in DCIS compared to adjacent normal tissue (Fig. 3B and Supplementary Fig. 2A). While SP21 staining in invasive breast cancer trended higher than in adjacent normal tissue, an observation validated in an independent cohort (Supplementary Fig. 2A), highest staining was observed in the DCIS lesions. With CX229, the highest COX2 expression was observed in histologically normal epithelium, with modest but progressive loss of COX2 expression in invasive cancer (Fig. 3B and Supplementary Fig. 2B). Importantly, these data show how clone selection for COX2 antibody may yield substantially different results and demonstrates the value of assessing COX2 expression using multiple antibody clones that delineate between S-nitrosylated and non-nitrosylated forms of COX2.

### Distinct intracellular localization of S-nitrosylated COX2

**Fig. 4:**
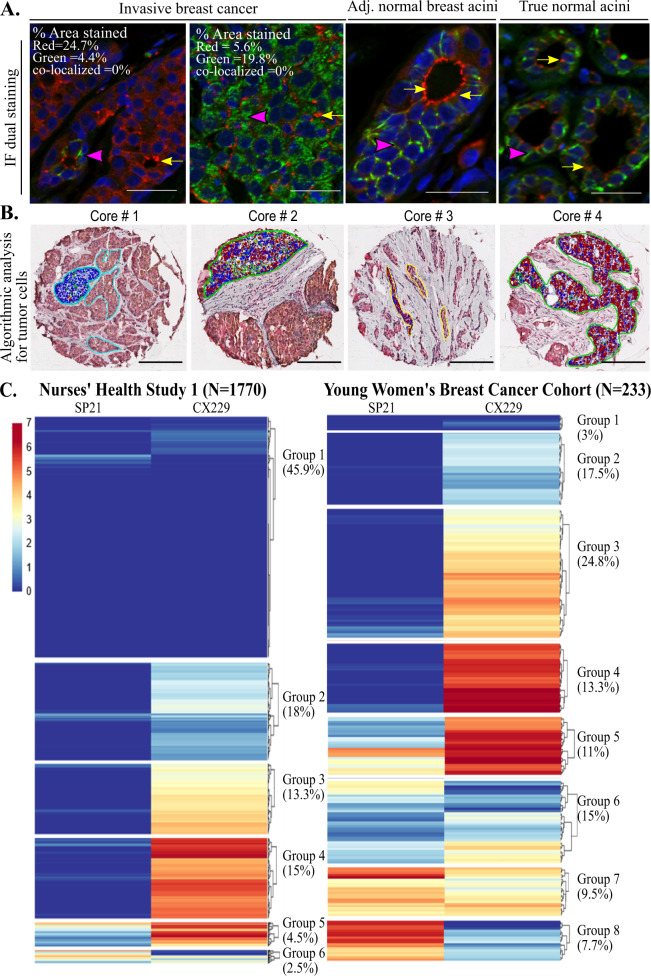
Variation of SP21 and CX229 expression in two breast cancer cohorts. **A** IF staining of two invasive cancer cases show predominant staining for either SP21 (far left panel) or CX229 (left middle panel) and absence of colocalization (SP21 = red, CX229 = green, and colocalization = yellow). Percent stained area for each clone and percent colocalization are listed within the images. Similarly, IF staining of adjacent normal (right middle panel) and true normal (far right panel) breast acini show SP21 (red signal) and CX229 (green signal) stain distinct cellular locations with minimal overlap (yellow signal). Pink arrow heads show intense localized staining for CX229. Yellow arrows show intense localized staining for SP21. Scale bar = 20 µm. **B** TMA cores with dual staining for SP21 and CX229 were annotated for tumor epithelium. The algorithmic analyses of positive staining for SP21 (red), CX229 (green), colocalization (yellow), and negative (blue) is shown. Scale bar is 100 µm. **C** Hierarchical clustering analysis for SP21 and CX229, assessed independently for each breast cancer cohort using RStudio, shows cohorts separate into distinct groups. NHS1 breast tumor cores (*N* = 8612, 1770 cases) clustered into six COX2 expression groups with the largest group (*n* = 3961, 45.9%), exhibiting very low expression for both SP21 and CX229. Groups 2 (*n* = 1580, 18%), 3 (*n* = 1154, 13.3%), and 4 (*n* = 1308, 15%), were defined by cores with low, medium, and high expression of CX229, respectively, and very low SP21 expression. Cluster 5 (*n* = 389, 4.5%) had medium level of SP21 expression and high CX229 expression, and cluster 6 (*n* = 220, 2.5%) contained cores with medium expression of SP21, but low expression of CX229 (left panel). The young women breast cancer study clustered into eight groups with cluster 1 (*n* = 7, 1.8%) with very low expression of both SP21 and CX229. Groups 2 (*n* = 34, 17.5%), 3 (*n* = 58, 24.8%), and 4 (*n* = 31, 13.3%) had very low SP21 and low, medium, and high CX229 expression, respectively. Groups 5 (*n* = 26, 11%) and 6 (*n* = 37, 15%) had high and low CX229 expression, respectively, with low to medium SP21 expression. Groups 7 (*n* = 22, 9.5%) and 8 (*n* = 18, 7.7%) had medium to high SP21 and low CX229 expression (right panel).

With the biochemical confirmation that SP21 and CX229 differentially recognize COX2 protein based on S-nitrosylation state, we next assessed whether these two forms of COX2 colocalize within cells. To obtain information about COX2 localization at a subcellular resolution, we performed dual immunofluorescence (IF) staining with SP21 and CX229, as well as with SP21 and CX294. We found that individual breast cancer cases could be dominated by SP21 signal (Fig. 4A, far left panel), CX229 signal (Fig. 4A, left middle panel), or both (data not shown). In addition, SP21 and CX294 dual-stained cases show nearly identical staining patterns to cases stained with SP21 and CX229 in invasive and normal adjacent breast acini, providing further evidence that CX229 and CX294 recognize the same COX2 epitope (Supplementary Fig. 4). Of note, there was virtually no overlap in cellular localization of SP21 and CX229/CX294, data consistent with SP21 and CX229/CX294 recognizing distinct forms of COX2 (Fig. 4A and Supplementary Fig. 4). Further in adjacent normal tissue, SP21 and CX229/CX294 stained distinct subcellular regions (Fig. 4A, right middle panel and Supplementary Fig. 4). Specifically, within acinar structures CX229 and CX294 dominantly stained lateral plasma membranes (Fig. 4A, right panel green signal and Supplementary Fig. 4), whereas SP21 primarily stained apical junctional regions, often in a punctate manner (Fig. 4A, right panel red signal and Supplementary Fig. 4). Similar staining patterns were found for SP21 and CX229 in true normal breast tissue (Fig. 4A, far right and Supplementary Fig. 5). These data are consistent with distinct trafficking, and function of S-nitrosylated and non-nitrosylated COX2 in adjacent and true normal breast tissue^49^.

### Variation of SP21 and CX229 staining in two large breast cancer cohorts

To further understand the inter-case variation between S-nitrosylated and non-nitrosylated COX2 expression in breast cancer, we stained two large breast cancer cohorts, the Nurse’s Health Study 1 (NHS1) and the University of Colorado Young Women’s Breast Cancer Translational Program (YWBCTP), which have ~2000 combined cases (Supplementary Table 3). We used an optimized dual SP21 and CX229 staining protocol where we confirmed that neither antibody order nor chromogen selection significantly impacted staining results (Supplementary Fig. 3). We found that ~95% of the COX2 signal comes from tumor cells compared to stromal cells, results consistent with previous reports^27,31^. To account for differences in stromal composition between cases, we restricted analyses to tumor cells by positively annotating tumor cell clusters followed by computer-assisted quantitation of antibody signal (Aperio ImageScope analysis software (Leica Biosystems, Vista, CA); Fig. 4B). To this end, SP21 and CX229 COX2 expression for each sample was compiled as a continuous variable, and hierarchical clustering was performed using R studio software. Independent K-mean clustering for SP21 and CX229 in both cohorts showed nearly identical cutoff values for positivity (SP21: NHS-2.9%, YWBCTP-2.8%; CX229: NHS-6.1%, YWBCTP-6.2%) indicating similar staining intensity, which permits comparisons between the two cohorts.

The NHS1 cohort is primarily composed of women diagnosed with breast cancer later in life, with an average age at diagnosis of 58 years, and consists of *N* = 8612 cores representing 1770 cases. By hierarchical clustering, the NHS1 breast tumor cores clustered into six COX2 expression groups with group 1 (45.9% of cores), the largest group, exhibiting very low expression for both SP21 and CX229 (Fig. 4C, group 1). Groups 2 (18% of cores), 3 (13.3% of cores), and 4 (15% of cores), were defined by cores with low, medium, and high expression of CX229, respectively, but very low SP21 expression. Group 5 (4.5% of cores) is defined by medium levels of SP21 expression and high CX229 expression. Finally, group 6 (2.5% of cores) contained cores with medium to high expression of SP21, but low expression of CX229. Since COX2 expression in the normal breast has been demonstrated to be hormone dependent^31,50^, we next assessed the expression of SP21 and CX229 in a cohort of young women’s breast cancer (YWBCTP, *N* = 233 cases) with an average age at diagnosis of 38 years. In the YWBCTP cohort, only 3% of cases had very low expression of CX229 and SP21 (Fig. 4D, group 1) compared to 45.9% of cores in the NHS1 cohort (Fig. 4C, group 1). In particular, SP21 was much higher in the YWBCTP cohort, which resulted in the formation of two additional groups with very high expression of SP21 in ~17% of cases (Fig. 4D, groups 7 and 8). In sum, COX2 expression, for both S-nitrosylated and non-nitrosylated COX2 varies widely between patients, with non-nitrosylated COX2 being more commonly expressed than the S-nitrosylated form. In addition, expression of both S-nitrosylated and non-nitrosylated COX2 is more common in the YWBCTP cohort of early-onset breast cancers compared to the older, NHS1 cohort.

## Discussion

In this study, we identify commonly used COX2 antibodies that differentially recognize distinct forms of COX2 based, at least in part, on posttranslational S-nitrosylation. Evidence of distinct cellular synthetic, trafficking, and functional pathways for these two forms of COX2 is suggested by nonoverlapping staining patterns in true normal, adjacent normal, and cancerous breast tissues. Similar staining patterns were observed in colon cancer, which supports relevance of these findings in the colon. Further, since COX2 biology is thought to be important in many cancer types and diseases, our discovery of S-nitrosylation state-specific COX2 antibody clones is likely to have broad impact. While we are the first to validate antibody reagents that distinguish COX2 based on its S-nitrosylation state, the impact of COX2 S-nitrosylation in the context of cancer remains to be determined.

In a small tissue cohort containing far adjacent normal, DCIS, and invasive breast cancer on a single slide, a tissue sample considered as a surrogate for disease progression^51^, we observed that S-nitrosylated COX2 is highest in DCIS lesions compared to adjacent normal breast tissue, with levels trending down in invasive cancer. These results are consistent with previously published work that showed HER2, a bona fide breast cancer oncogene, was most highly expressed in DCIS lesions^52^. Overall, high expression of S-nitrosylated COX2 in DCIS lesions and invasive cancer is an observation consistent with the established paradigm of COX2 as pro-tumorigenic^35–39^. However, using sequential tissue sections, we found that non-nitrosylated COX2 (CX229) was decreased between adjacent normal and invasive tumor, an observation we confirmed in an independent tissue cohort (Fig. 3B and Supplementary Fig. 2). Decreased COX2 staining pattern with disease progression has been previously reported^31,41,42^. While intriguing, these COX2 clone-specific results require validation in larger cohorts.

It is worth speculating as to why COX2 staining in adjacent normal breast tissue may differ so widely between studies. Considering that reproductive biology is reported to play a role in COX2 expression, one possible explanation could be due to the balance of premenopausal and postmenopausal women included in each study. It is worth noting that our disease progression cohorts were comprised entirely of premenopausal women. Previous studies show that COX2 is dynamically regulated in mammary gland tissue across a menstrual cycle, and induced by ovarian hormones in rodents^31,50^. In support of menopausal status impacting COX2 expression in human breast tissue, a study of breast biopsies from healthy women showed that COX2 expression is dynamically regulated across a reproductive cycle^53^. Further, previously published studies revealed that COX2 expression in healthy, premenopausal women was highly variable, with positive CX229 or CX294 staining ranging from undetectable to high^31,42,54^. Our observations reported here from over 2000 breast cancer cases showed that early-onset breast cancer has increased expression of both forms of COX2 compared to postmenopausal cases (Fig. 4C), which is consistent with hormonal regulation of COX2 in cancerous tissues as well. Additional research is needed to better understand the role of menopausal status in COX2 biology and its S-nitrosylation state.

COX2 S-nitrosylation is dependent on NO biology, and data from the NO field provides strong rational for pursuing a potential role for COX2 S-nitrosylation in breast cancer. In endothelial and neuronal cells, NO is produced via expression of the NO synthases eNOS and nNOS, which regulate physiologic vasodilation and neuronal signaling, respectively^55^. Evidence for an inducible form of NO synthase (iNOS) was first reported in macrophages^56^, and led to the discovery of NO as a principal regulator of tissue inflammation^55^ with likely roles in cancer^55,57^. For example, in triple-negative breast cancer (TNBC) cell lines, defined as ER, PR, and Her-2 negative, iNOS signaling promotes stem-like properties and metastatic potential^58^. Further, iNOS blockade as a single agent reduced TNBC growth, metastasis^59^, and enhanced efficacy of chemotherapy in xenograft models^60^. Importantly, these preclinical studies define a novel, NO-centric path toward the possible treatment of aggressive TNBC. Interrogating COX2 S-nitrosylation by breast cancer subtype, grade, and stage is a potentially fruitful next step for understanding COX2 as a biomarker of breast cancer risk, as well as a therapeutic target.

Evidence that S-nitrosylation increases COX2 activity has been demonstrated using in vitro pathogen^47^ and neurotoxicity^48^ models, and in an in vivo model of myocardial infarction^61^. Further, studies demonstrate that iNOS inhibitors can block COX2 activity and its downstream pathogenic sequela, demonstrating a synergistic interaction between these two major inflammatory systems^62,63^. Consistent with NOS2 and COX2 inflammatory pathway cross talk in human breast cancer, a recent report finds co-expression of iNOS and COX2 predicts poor survival in breast cancer patients, and animal modeling confirms survival benefit with dual targeting of iNOS and COX2^62^. Thus, our work identifying antibodies that distinguish COX2 based on nitrosylation state in human tissue highlights the need for future investigations into the role that COX2 S-nitrosylation plays in breast cancer risk, progression, and outcomes.

Main strengths of our study include the use of multiple, independent methods to investigate COX2 specificity of four different αCOX2 antibody clones; the use of robust biochemical approaches to demonstrate dependency of S-nitrosylation state on COX2 antibody recognition; and the inclusion of over 2000 breast cancer cases to assess dual COX2 staining. One limitation of our study is that assessment of COX2 levels in adjacent normal, DCIS, and invasive cancers is based on a limited number of cases.

To conclude, we find that commonly utilized antibodies directed against COX2 can distinguish between S-nitrosylated and non-nitrosylated forms of COX2. Further, we find that S-nitrosylated and non-nitrosylated COX2 have distinct subcellular distributions in both normal and cancer tissues, providing evidence for distinct synthetic, trafficking, and functional pathways^49^. As a result, previous work relying on COX2 IHC to define associations between COX2 expression and cancer parameters should be reviewed in light of these findings. Likewise, future COX2 studies should be designed with multiple antibody clones to detect both S-nitrosylated and non-nitrosylated forms of COX2. How both forms of COX2 are regulated, which tissue compartments express COX2 (e.g., epithelial, endothelial, and immune), what subcellular locations they occupy, and how subcellular localization impacts COX2 function remain important, unanswered questions.

## Methods

### Ethics

FFPE human breast and colon tissue for this study was approved by the BWH/Harvard Cohorts Biorepository and Institutional Review Boards at Colorado Multiple Institution Review Board, and Oregon Health and Science University. Written informed consent was given by participants when required.

### Human tissues

De-identified FFPE cases of breast (*n* = 52) and colon (*n* = 53) cancer TMAs and breast cancer cases (*n* = 2019) from the NHS1^64^ were obtained from the Channing Laboratory, Brigham and Women’s Hospital, Massachusetts. Young women’s FFPE breast cancer cases were acquired from the YWBCTP at the University of Colorado (*n* = 233). Breast tissue sections with adjacent normal, DCIS, and invasive ductal carcinoma on a single slide were obtained from Kaiser Permanente Northwest (*n* = 10). In cases with cancer, normal adjacent lobules, as determined by a pathologist, were analyzed at minimum of 4 mm away from the tumor. The far adjacent normal lobules were at least 1 cm away from cancer tissue. A total of 233 YWBCTP and 1770 NHS1 cases were evaluated for dual COX2 IHC stain after exclusion of one entire TMA slide (249 cases) from the NHS1 cohort. The control cores for this TMA slide displayed staining several standard deviations above the average for the study, resulting in exclusion from analysis.

### Antigenic regions used for antibody generation

COX2 protein sequence for human (P35354), rat (P35355), and mouse (Q05769) were aligned with Uniprot (http://www.uniprot.org/). Antigenic peptide sequences used to generate each COX2 clone were obtained from the respective manufacturers and examined for differences in species and posttranslational modification sites.

### Immunoblotting

Recombinant human COX1 protein (Abcam, Ab-198643, 4 ng), human COX2 protein (Cayman Chemical, 60122, 4 ng), and 25 µg cell line lysates in RIPA buffer were separated by WES automated gel electrophoresis system (Protein Simple, San Jose, CA). Cell lines were procured from authenticated sources: mouse BRAFV600E melanoma (Wt) and COX1/2 CRISPR targeted subline^44^; human HCA-7 colon cancer (Sigma Aldrich #02091238) and human HCT-15 colon cancer (ATCC, #CCL-225). Primary antibodies and working concentrations were: COX2 SP21 clone (Thermo Fisher Scientific #RM-9121, at 25 ng/µL), COX2 CX229 clone (Cayman Chemical #160112, at 25 ng/µL), COX2 CX294 clone (Agilent Dako #M3617, at 25 ng/µL) and COX2 D5H5 clone (Cell Signaling Technology #12282, at 25 ng/µL), COX1 (Cell Signaling Technology #4841, at 25 ng/µL), and GAPDH (14C10 clone, Cell Signaling Technology #2118, at 2 ng/µL). For HRP-conjugated secondary antibodies and detection; anti-rabbit (Protein Simple #042-206, RTU) or anti-mouse (Protein Simple #042-205, RTU) were utilized, followed by chemiluminescent substrate (Protein Simple #PS-CS01, Luminol-S, Peroxide). Signal was detected using the WES System camera. Immunoblot electrophoreograms were analyzed by Compass Software (Protein Simple, San Jose, CA). We confirm all blots derive from the same experiment and were processed in parallel.

### S-nitrosylation and chemical de-nitrosylation

S-nitrosylation of proteins was detected by western blot using an S-nitrosylation specific antibody (HY8E12 clone, Abcam # 94930, 1:20) or the Pierce S-Nitrosylation Western Blot detection kit (Thermo Fisher Scientific # 90105). De-nitrosylation of proteins was performed using either 330 mM (low) or 1 M (high) Na ascorbate in HENS buffer (Thermo Fisher Scientific #90106), as previously described^47^^,48^.

### Immunohistochemical staining of FFPE tissues

Four µm sections of FFPE tissue were stained for single or dual COX2 IHC, or dual COX2 IF. Positive control breast cancer tissue has been previously described^31^. Detailed protocols for staining are outlined in Supplementary Table 4. COX2 antibody clones were SP21 (Thermo Fisher scientific #RM-9121), CX229 (Cayman #160112), and CX294 (Agilent Dako #M3617). Secondary antibodies and chromogens were Envision+ HRP detection (Agilent #K4001 and #K4003) followed by 3,3′-diaminobenzidine (Agilent #K3468), or alkaline phosphatase detection (Enzo Life Sciences #ACC110-0150) followed by Warp Red (Biocare #WR806) for IHC staining and Alexa Fluor antibodies (Invitrogen #A11029 and #A21245) for IF staining. IHC and IF stained slides were scanned using Aperio ScanScope AT (Leica Biosystems, Vista, CA) and Apotome (Zeiss, Jena, Germany) microscopes, respectively. IHC signal data were captured and quantified using Aperio ImageScope analysis software (Leica Biosystems, Vista, CA)^31^. All data acquisition was performed by investigators who were blinded to study group.

### Hierarchical and K-means clustering of SP21 and CX229 expression

Percent area stained for SP21 and CX229 dual-stained FFPE tissue from the NHS1 and YWBCTP cohorts were separately subjected to hierarchical and K-means clustering, and optimal cluster numbers were obtained. Hierarchical clustering was performed using R studio software. For K-means clustering, the lowest expression group was identified as the distribution containing the negative stained group above which all values would be considered positive^65,66^.

### Statistical analysis

Comparisons for far/near adjacent normal, DCIS, and invasive cancer were done on GraphPad Prism 8 software using the two tailed *t* test, with significance at *P* value of < 0.05. Comparisons of clinical characteristics of the NHS1 and YWBCTP cohorts was performed using chi-squared test on GraphPad Prism 8 software.

### Reporting summary

Further information on research design is available in the Nature Research Reporting Summary linked to this article.

## Supplementary information

Supplementary Data

Reporting Summary Checklist

## Data Availability

The data generated and analyzed during the current study are publicly available in the figshare repository: 10.6084/m9.figshare.13009985^67^. The clinical data that support the findings of this study are available from the corresponding author upon reasonable request, as described in the data record above.
