# Supplementary material for: S-nitrosylated and non-nitrosylated COX2 have differential expression and distinct subcellular localization in normal and breast cancer tissue
Source: NPJ Breast Cancer. 2020 Nov 24;6:62. doi: 10.1038/s41523-020-00204-6 (PMC7686348; doi:10.1038/s41523-020-00204-6)
Supplement: Supplementary file 1 — Supplementary Data [file 41523_2020_204_MOESM1_ESM.pdf]

Supplementary Table 1: COX-2 expression in Breast TMA comparing COX2 clones CX229 and CX294

**TMA Staining Results**	**COX-2 Clones**	**COX-2 Positive Breast TMA (n=56)**
Dual Positive COX-2	CX229+/CX294+	53 (95%)
Positive for CX229 only	CX229+/CX294-	0
Positive for CX294 only	CX229-/ CX294+	0
Dual negative COX-2	CX229-/ CX294 -	3 (5%)

Supplementary Table 2: COX-2 expression in Breast and Colon TMA comparing clones SP21 and CX229

TMA Staining Results	COX-2 Clones	COX-2 Positive Breast TMA (n=52)	COX-2 Positive Colon TMA (n=53)
Non-nitrosylated COX-2 only	CX229+/SP21-	18 (35%)	23 (43%)
Dual positive COX-2	CX229+/SP21+	29 (55%)	21 (40%)
S-nitrosylated COX-2 only	CX229-/SP21+	1 (2%)	0 (0%)
Dual negative COX-2	CX229-/SP21-	4 (8%)	9 (17%)

Supplementary Table 3: Clinical characteristics of NHS 1 and YWBC cohorts

Clinical characteristics	Nurses' Health Study 1	Young Women Breast Cancer	P value (chi square test)
**Overall N**	1770 cases	233 cases	
**Age at dx, mean**	57.8	38.24	
**Stage**			
1	885 (50%)	67 (28.7%)	
2	600 (33.9%)	97 (41.6%)	<0.0001
3	250 (14.1%)	52 (22.3%)	
4	35 (2%)	15 (6.52%)	
**Grade**			
1	303 (17.1%)	22 (9.4%)	
2	935 (52.8%)	86 (36.9%)	<0.0001
3	416 (23.5%)	109 (46.8%)	
Missing	116 (6.6%)	16 (6.9%)	
**Tumor size (cm)**			
≤1	439 (24.6%)	30 (12.9%)	
1.1-2.0	682 (38.3%)	73 (31.4%)	<0.0001
2.1-4.0	481 (27.1%)	65 (27.9%)	
>4.0	164 (9.8%)	53 (22.7%)	
Missing	4 (0.2%)	12 (5.1%)	
**ER status**			
Positive	1361 (76.9%)	142 (60.9%)	
Negative	404 (22.8%)	87 (37.3%)	<0.0001
Missing	5 (0.3%)	4 (1.8%)	
**PR status**			
Positive	1137 (64.2%)	135 (57.9%)	
Negative	625 (35.3%)	93 (39.9%)	0.1156
Missing	8 (0.5%)	5 (2.1%)	
**HER2 status**			
Positive	195 (11.0%)	67 (28.7%)	<0.0001
Negative	1546 (87.4%)	147 (63%)	
Missing	29 (1.6%)	19 (8.3%)	
**Hormone therapy**			
Yes	837 (47.4%)	79 (33.9%)	
No	433 (24.6%)	67 (28.8%)	0.0047
Missing	496 (28%)	87 (37.3%)	
**Radiation**			
Yes	545 (30.7%)	90 (38.6%)	
No	735 (41.5%)	55 (43.5%)	<0.0001
Missing	490 (27.8%)	88 (37.8%)	
**Chemotherapy**			
Yes	492 (27.8%)	137 (58.9%)	
No	772 (43.6%)	19 (8.1%)	<0.0001
Missing	506 (28.6%)	77 (33%)	

Supplementary Table 4: The methodological details for IHC and IF staining

Stain/single or dual	Antigen retrieval	Peroxidase and Protein Blocks	Primary Antibody	Secondary Antibody	Chromogen
**IHC – SP21 stain**	125ºC under pressure for 5 mins with TRS (Dako #S1699)	Peroxidase block (1ml 3% H_2O_2 + 9 ml methanol) for 10 mins<br>Protein block (Biocare # BS966L) for 10 mins	SP21 clone (Thermoscientific # RM-9121) 1:200 for 60 mins	Envision + HRP antibody (Dako # K4003 rabbit) for 30 mins	DAB (Dako # K3468) for 10 mins
**IHC-CX229 stain**	125ºC under pressure for 5 mins with TRS (Dako #S1699)	Peroxidase block (1ml 3% H_2O_2 + 9 ml methanol) for 10 mins<br>Protein block (Biocare # BS966L) for 10 mins	CX229 clone (Cayman, # 160112) 1:250 for 60 mins	Envision + HRP antibody (Dako # K4001 mouse) for 30 mins	DAB (Dako # K3468) for 10 mins
**IHC-CX294 stain**	125ºC under pressure for 5 mins with TRS (Dako #S1699)	Peroxidase block (1ml 3% H_2O_2 + 9 ml methanol) for 10 mins<br>Protein block (Biocare # BS966L) for 10 mins	CX294 clone (Agilent # M3617) 1:50 for 60 mins	Envision + HRP antibody (Dako # K4001 mouse) for 30 mins	DAB (Dako # K3468) for 10 mins
**IHC- Dual SP21 and CX229 stain**	125ºC under pressure for 5 mins with TRS (Dako #S1699)	*1st antibody stain*: Peroxidase (1ml 3% H_2O_2 + 9 ml methanol) for 10 mins<br>Protein block (Biocare # BS966L) for 10 mins<br>*2nd antibody stain*: Protein block (Biocare # BS966L) for 10 mins	CX229 clone (Cayman, #160112) 1:250for 60 mins	Envision + HRP antibody (Dako # K4001mouse) for 30 mins	DAB (Dako # K3468) for 10 mins

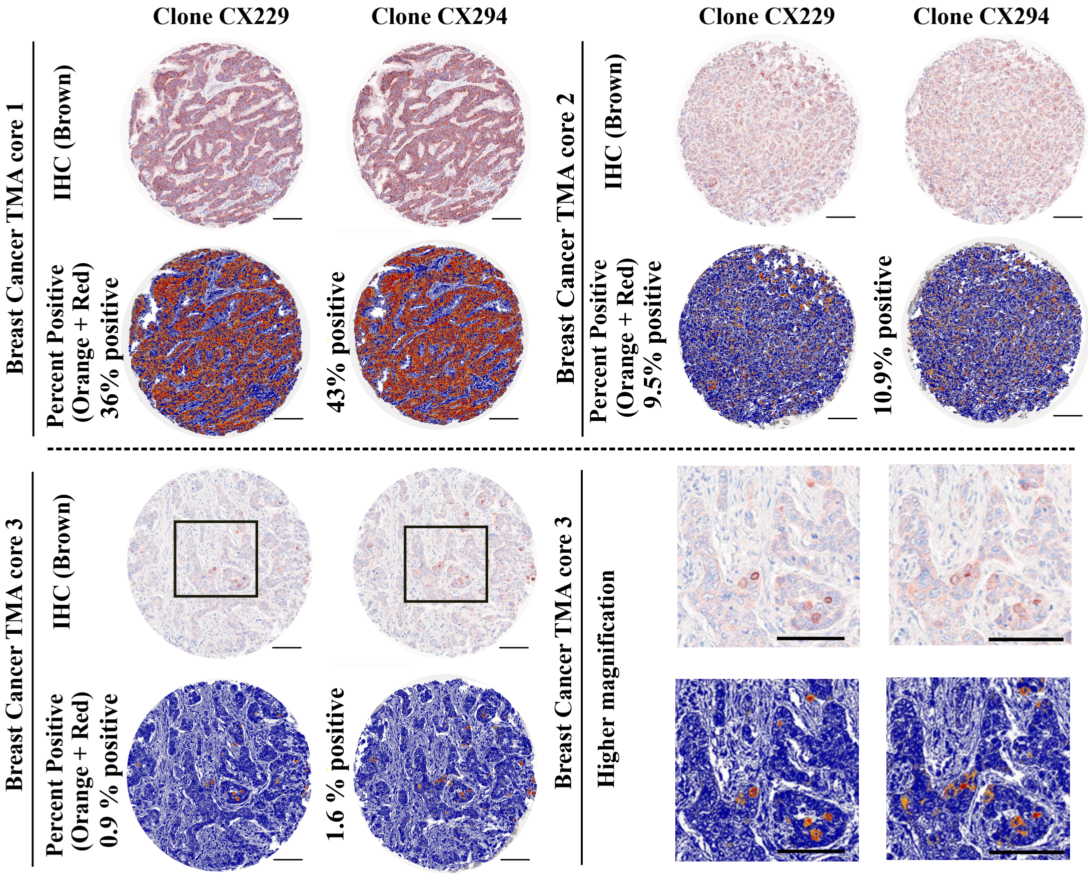

Supplementary Figure 1: TMA cores from breast cancer cases stained for CX229 and CX294 have similar staining intensity, ranging from high (core #1), medium (core #2) or low (core #3). Higher magnification of core #3 (black box) shows that CX229 and CX294 have similar specificity in staining the same cells on sequential sections. Algorithmic analysis for each TMA core shows COX2 positive signal (orange and red) compared to negatively stained tissue (blue). Scale bar for all images is 100µm.

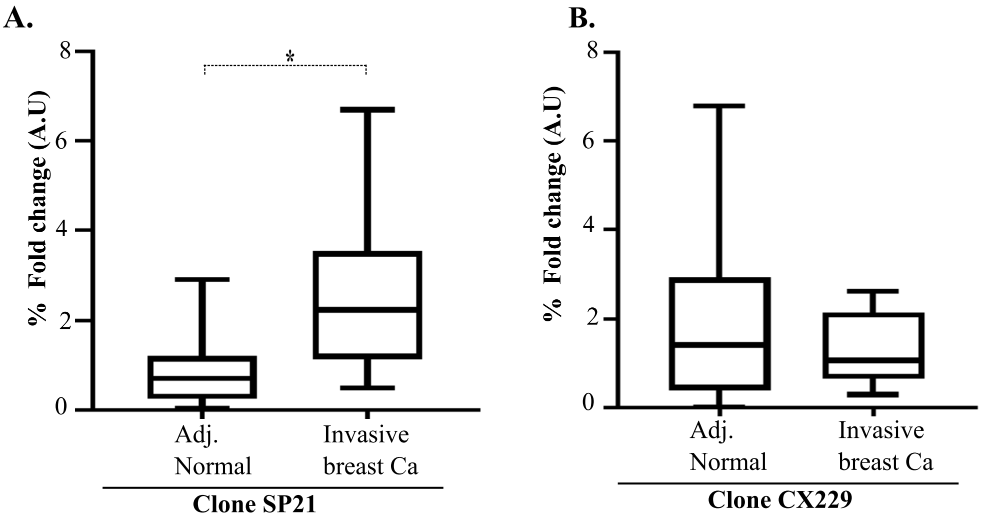

Supplementary Figure 2. SP21 and CX229 clones show inverted staining patterns between adjacent normal and invasive cancer in Kaiser Pacific North West (KPNW) cases. A. SP21 staining intensity was low in adjacent normal breast tissue and increased in invasive breast cancer (n = 10, P value: *≤0.05). B. CX229 (n = 10) shows highest COX2 expression in normal adjacent breast epithelium with a trend towards decreased COX2 expression in invasive breast cancer.

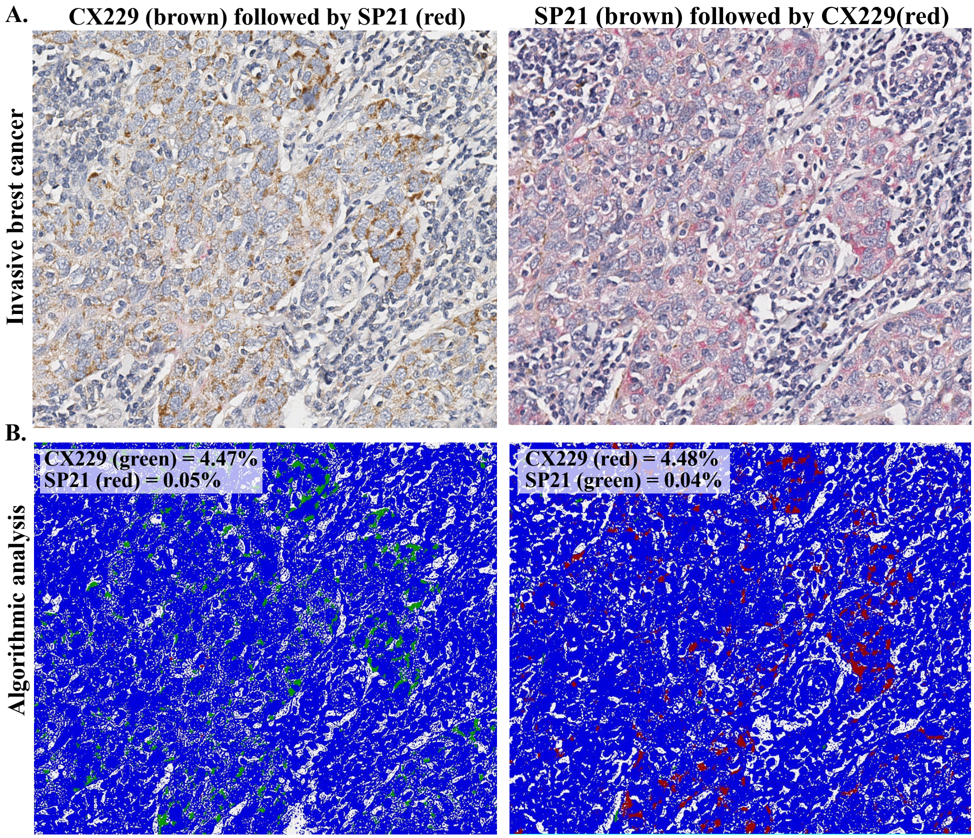

Supplementary Figure 3. A. Dual IHC stained images of SP21 and CX229 show similar staining patterns when the order of antibodies and chromogens were switched during staining process. B. Algorithmic analysis of the images in part A of this figure (upper panel) revealed 4.47% CX229 (green) and 0.05% SP21 (red) expression (left, lower panel). With reversed antibody order, there was 4.48% CX229 (red) and 0.04% SP21 (green) expression (right, lower panel).

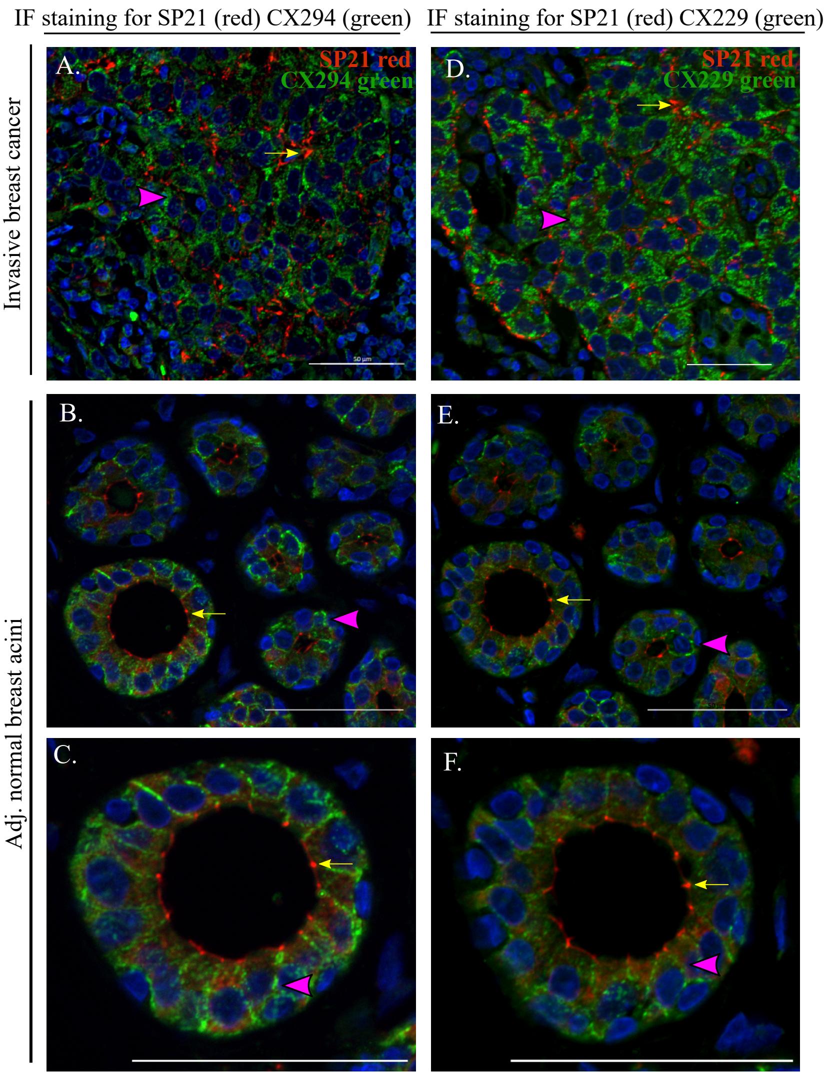

Supplementary Figure 4. IF staining of serial sections of an invasive cancer case and adjacent normal breast acini show distinct sub-cellular localization of SP21, CX294 and CX229. The left column shows dual stains of SP21 and CX294 with minimal co-localization (SP21 = red, CX294 = green, co-localization = yellow). The right column shows dual stains of SP21 and CX229 with minimal co-localization (SP21 = red, CX294 = green, co-localization = yellow). Pink arrow heads show intense localized staining for CX294/CX229. Yellow arrows show intense localized staining for SP21. Scale bar for all images is 50µm.

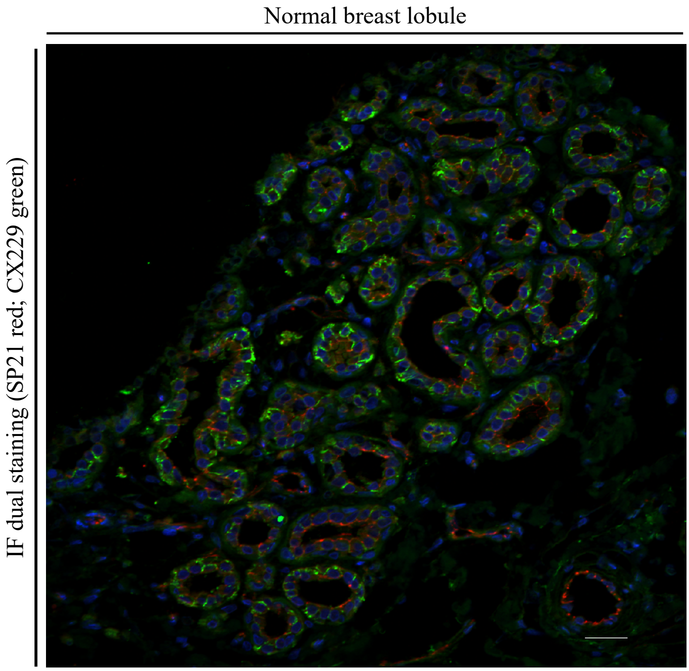

Supplementary Figure 5. IF staining of a true normal lobule with breast acini show distinct sub-cellular localization of SP21 and CX229. The dual stains of SP21 and CX229 have minimal co-localization (SP21 = red, CX294 = green, co-localization = yellow) but have heterogeneous expression in each acini. Scale bar for all images is 30µm.
