# Supplementary material for: S-nitrosylated and non-nitrosylated COX2 have differential expression and distinct subcellular localization in normal and breast cancer tissue
Source: NPJ Breast Cancer. 2020 Nov 24;6:62. doi: 10.1038/s41523-020-00204-6 (PMC7686348; doi:10.1038/s41523-020-00204-6)
Supplement: Supplementary file 2 — Reporting Summary Checklist [file 41523_2020_204_MOESM2_ESM.pdf]

nature research

Corresponding author(s): Pepper Schedin

Last updated by author(s): Apr 13, 2020

Reporting Summary

Nature Research wishes to improve the reproducibility of the work that we publish. This form provides structure for consistency and transparency in reporting. For further information on Nature Research policies, see Authors & Referees and the Editorial Policy Checklist.

Statistics

For all statistical analyses, confirm that the following items are present in the figure legend, table legend, main text, or Methods section.

n/a	Confirmed	
☐	☒	The exact sample size (*n*) for each experimental group/condition, given as a discrete number and unit of measurement
☐	☒	A statement on whether measurements were taken from distinct samples or whether the same sample was measured repeatedly
☐	☒	The statistical test(s) used AND whether they are one- or two-sided<br>*Only common tests should be described solely by name; describe more complex techniques in the Methods section.*
☐	☒	A description of all covariates tested
☒	☐	A description of any assumptions or corrections, such as tests of normality and adjustment for multiple comparisons
☐	☒	A full description of the statistical parameters including central tendency (e.g. means) or other basic estimates (e.g. regression coefficient) AND variation (e.g. standard deviation) or associated estimates of uncertainty (e.g. confidence intervals)
☐	☒	For null hypothesis testing, the test statistic (e.g. *F*, *t*, *r*) with confidence intervals, effect sizes, degrees of freedom and *P* value noted<br>*Give P values as exact values whenever suitable.*
☒	☐	For Bayesian analysis, information on the choice of priors and Markov chain Monte Carlo settings
☐	☒	For hierarchical and complex designs, identification of the appropriate level for tests and full reporting of outcomes
☒	☐	Estimates of effect sizes (e.g. Cohen's *d*, Pearson's *r*), indicating how they were calculated

Our web collection on statistics for biologists contains articles on many of the points above.

Software and code

Policy information about availability of computer code

Data collection	Commercial Aperio ImageScope analysis software V9(Leica Biosystems, Vista, CA) was used in data collection
Data analysis	Commercial Graph Pad prism version 8.0 was used for the statistical analysis of data in the manuscript

For manuscripts utilizing custom algorithms or software that are central to the research but not yet described in published literature, software must be made available to editors/reviewers. We strongly encourage code deposition in a community repository (e.g. GitHub). See the Nature Research guidelines for submitting code & software for further information.

Data

Policy information about availability of data

All manuscripts must include a data availability statement. This statement should provide the following information, where applicable:

- Accession codes, unique identifiers, or web links for publicly available datasets
- A list of figures that have associated raw data
- A description of any restrictions on data availability

The data that support the findings of this study are available from the corresponding author upon reasonable request.

Field-specific reporting

Please select the one below that is the best fit for your research. If you are not sure, read the appropriate sections before making your selection.

☒ Life sciences ☐ Behavioural & social sciences ☐ Ecological, evolutionary & environmental sciences

For a reference copy of the document with all sections, see nature.com/documents/nr-reporting-summary-flat.pdf

October 2018

Life sciences study design

All studies must disclose on these points even when the disclosure is negative.

Sample size	De-identified FFPE cases of breast (n=52) and colon (n=53) cancer tissue microarrays (TMAs) and breast cancer cases (n=2019) from the Nurses' Health Study-1 (NHS1) were obtained from the Channing Laboratory, Brigham and Women's Hospital, Massachusetts.<br>De-identified cases from Young women's FFPE breast cancer cases were acquired from the Young Women's Breast Cancer Translational Program (YWBCTP) at the University of Colorado (n = 233).<br>De-identified cases from Breast tissue sections with adjacent normal, ductal carcinoma in situ (DCIS) and invasive ductal carcinoma on a single slide were obtained from Kaiser Permanente Northwest (KPNW) (n=10).
Data exclusions	The control cores for one TMA slide (n=249 cases) from NHS1 displayed staining several standard deviations above the average for the study, resulting in exclusion from the analysis.
Replication	The data for COX2 clones SP21, CX229 and CX294 has been replicated and reproduced using multiple methods including western blot, IHC and IF staining . The data is included in the manuscript .
Randomization	Tissue samples for our study were received from prospective or retrospective observational studies thus patient randomization is not relevant to our study. For technical randomization of IHC stain for every staining run the same control tissue was used to serve as an internal control for inter run assay variation.
Blinding	All investigators who performed the staining and data analysis were blinded to study group and clinical information of the participants.

Reporting for specific materials, systems and methods

We require information from authors about some types of materials, experimental systems and methods used in many studies. Here, indicate whether each material, system or method listed is relevant to your study. If you are not sure if a list item applies to your research, read the appropriate section before selecting a response.

Materials & experimental systems

n/a	Involved in the study	
☐	☒	Antibodies
☐	☒	Eukaryotic cell lines
☒	☐	Palaeontology
☒	☐	Animals and other organisms
☐	☒	Human research participants
☒	☐	Clinical data

Methods

n/a	Involved in the study	
☒	☐	ChIP-seq
☒	☐	Flow cytometry
☒	☐	MRI-based neuroimaging

Antibodies

Antibodies used	Recombinant human COX1 protein (Abcam, Ab-198643, 4ng); human COX2 protein (Cayman Chemical, 60122, 4ng); COX2 SP21 clone (Thermo Fisher Scientific # RM-9121, AB_720732, at 25ng/uL); COX2 CX229 clone (Cayman Chemical # 160112,AB_10078980, at 25ng/uL); COX2 CX294 clone (Agilent Dako # M3617, at 25ng/uL); COX2 D5H5 clone (Cell Signaling Technology # 12282,AB_2571729 , at 25ng/uL); COX-1 (Cell Signaling Technology # 4841, AB_2084807, at 25ng/uL); GAPDH (14C10 clone, Cell Signaling Technology #2118, AB_561053, at 2ng/uL); S-nitrosylation specific antibody (HY8E12 clone, Abcam # 94930, AB_10697568); anti-rabbit (Protein Simple # 042-206, RTU) or anti-mouse (Protein Simple# 042-205, RTU); Envision+ HRP detection (Agilent # K4001 (AB_2827819), # K4003 (AB_2630375)]
Validation	Our manuscript describes intensive validation for COX-2 antibody clones (CX229, SP21, CX294. D5H5) using Western blotting and /or immunohistochemistry. All Cell Signaling Technology antibodies are certified as meeting the quality control standards of Cell Signaling Technology per certificates of analysis using authentication methods such as Western blot analyses with siRNA knockdown, use of positive and negative tissue, cell extracts, and xenografts with known target expression, and use of blocking peptides where possible. For all abcam products, application notes include validated applications per Western blotting and tissue microarray staining, and recommended starting dilutions, with optimal conditions determined by end-user. Cayman recombinant COX2 and COX1 purity was determined by manufacturer using SDS-Page.

Eukaryotic cell lines

Policy information about cell lines

Cell line source(s)	HCA7- Colony 29 - purchased directly from Sigma Aldrich (Cat no 02091238), HCT-15 purchased directly from ATCC (Cat#CCL-225), BRAFV600E Melanoma WT and CRISPRKO Cell Lines obtained directly from originating investigators Dr. Santiago Zelenay (CRUK) and Dr. Caetano Reis E Sousa (CRICK).
Authentication	HCA7-Colony 29 Profiled by STR-DNA profiling, obtained from Sigma-Aldrich in conjunction with the European Collection of

Authentication	Authenticated Cell Culture (ECACC), HCT-15 validated at ATCC by STR, DNA fingerprint profiling and Karotyping. BRAFV600E cell lines that were COX1/COX2 WT and KO were obtained directly from originating investigators . We authenticated COX1/2 expression levels in these cell lines by Western blot analyses using several validated antiCOX1 and antiCOX2 antibodies and recombinant purified protein as positive controls.
Mycoplasma contamination	HCA7 colon 29 and HCT-15 cell lines were provided from vendors, verified free of mycoplasma. BRAFV600E samples were not tested for mycoplasma
Commonly misidentified lines (See ICLAC register)	No commonly misidentified cell line was used in our study

Human research participants

Policy information about studies involving human research participants

Population characteristics	The study includes Formalin-fixed paraffin-embedded (FFPE) human breast tissue samples from women aged >20 years and were diagnosed with breast cancer. The colon TMA samples are from both men and women who were diagnosed with colon cancer.
Recruitment	Participants were not recruited to this study. This study was retrospectively conducted on the Formalin-fixed paraffin-embedded (FFPE) human breast and colon tissue samples collected previously by the Nurses's Health Study, Young Women's Breast cancer Translational Program and NW Kaiser Permanente as part their study protocols.
Ethics oversight	Formalin-fixed paraffin-embedded (FFPE) human breast and colon tissue for this study was approved by the BWH/Harvard Cohorts Biorepository and Institutional Review Boards at Colorado Multiple Institution Review Board (COMIRB), and Oregon Health and Science University (OHSU).

Note that full information on the approval of the study protocol must also be provided in the manuscript.
